# Prevalence, Awareness, Treatment, and Control of Hypertension in the United Arab Emirates: A Systematic Review and Meta-Analysis

**DOI:** 10.3390/ijerph182312693

**Published:** 2021-12-02

**Authors:** Akshaya Srikanth Bhagavathula, Syed Mahboob Shah, Elhadi Husein Aburawi

**Affiliations:** 1 Institute of Public Health, College of Medicine and Health Sciences, UAE University, Al Ain 17666, Abu Dhabi, United Arab Emirates; 201890132@uaeu.ac.ae (A.S.B.); syeds@uaeu.ac.ae (S.M.S.); 2 Department of Pediatrics, College of Medicine and Health Sciences, UAE University, Al Ain 17666, Abu Dhabi, United Arab Emirates

**Keywords:** hypertension, blood pressure, prevalence, awareness, control, meta-analysis

## Abstract

Background: Evidence for the prevalence, awareness, treatment, and control of hypertension in the United Arab Emirates (UAE) is limited. A systematic review and meta-analysis were conducted to summarize the existing knowledge regarding the prevalence, awareness, treatment, and control of hypertension in the UAE. Methods: We searched PubMed/MEDLINE, Embase, Scopus, and Google Scholar using prespecified medical subject handling (MeSH) terms and text words to identify the relevant published articles from 1 January 1995 to 31 August 2021. Population-based prospective observational studies conducted among healthy adult subjects living in the UAE and that defined hypertension using the guidelines-recommended blood pressure (BP) cut-offs ≥ 130/80 mmHg or ≥ 140/90 mmHg were considered. Results: Of 1038 studies, fifteen cross-sectional studies were included for data extraction involving 139,907 adults with a sample size ranging from 74 to 50,138 and with cases defined as blood pressure ≥ 140/90 mmHg. The pooled prevalence of hypertension was 31% (95% confidence interval (CI): 27–36), and a higher prevalence was observed in Dubai (37%, 95% CI: 28–45) than in the Abu Dhabi region (29%, 95% CI: 24–35) and in multicenter studies (24%, 95% CI: 14–33). The level of awareness was only 29% (95% CI: 17–42), 31% (95% CI: 18–44) for treatment, and 38% (95% CI: 19–57) had controlled BP (< 140/90 mmHg). Conclusion: This study revealed a high prevalence of hypertension with low awareness and suboptimal control of hypertension. Multifaceted approaches that include the systematic measurement of BP, raising awareness, and improving hypertension diagnoses and treatments are needed.

## 1. Introduction

Hypertension, or high blood pressure (BP), is one of the most important risk factors and is a leading preventable cause of cerebrovascular, cardiovascular, and renal morbidity and mortality [1]. In 2013, the World Health Organization (WHO) set a goal to reduce hypertension prevalence by 25% by 2025, by reducing salt consumption and other public health measures [2].

Studies have found a doubling risk of ischemic heart disease and stroke with every 20 mmHg and 10 mmHg increases in systolic and diastolic BP, respectively [3]. In addition, the observational results indicated that each 10-mmHg increase in systolic blood pressure (SBP) was associated with a 45% higher risk of ischemic heart disease and stroke (65%) in those aged 55–64 years [4]. Hypertension can be diagnosed and treated early through population-based screening, and control is possible through behavioral and lifestyle changes such as decreased tobacco use, alcohol consumption, salt intake, physical activity, stress, and obesity.

Several international studies have reported global and regional variations in the prevalence, awareness, treatment, and control of hypertension [5,6,7,8]. For example, in the Middle East, findings from the Prospective Urban Rural Epidemiology (PURE) study showed that the prevalence of hypertension was high (33%) and that the lack of awareness (49%) was also high, while BP control was only 19% [8]. Although several studies investigated the burden of hypertension in the Middle Eastern countries [8,9,10,11,12], only a few reported the prevalence, awareness, treatment, and control rates. Individual studies have shown variation in the prevalence of hypertension in the UAE. For example, Hajat et al. [13] and Yousufali et al. [14] reported a prevalence rate of 23.1% and 52%, respectively.

Information on hypertension prevalence, awareness, treatment, and control is necessary to provide a baseline for monitoring, and for the development of new strategies for improving, hypertension control and resource planning. Although the early prevention of and screening for hypertension is necessary, there is evidence that suggests that there is poor disease awareness and BP control in the UAE [14,15,16]. Despite individual studies on adults in the UAE, there has been no comprehensive study on hypertension prevalence, awareness, treatment, and control. Moreover, previous systematic reviews conducted in Arabian populations did not consider studies from the UAE [17,18]. Thus, a systematic review and meta-analysis was conducted to investigate the prevalence, awareness, treatment, and control of hypertension in the UAE population.

## 2. Materials and Methods

The study protocol has been registered in the international registry of systematic reviews: PROSPERO (CRD42019141478). The systematic review and meta-analysis were both conducted following the updated Preferred Reporting Items for Systematic Review and Meta-analysis (PRISMA) statement 2020 [19].

### 2.1. Search Strategy

The literature search was conducted with PubMed/MEDLINE, Scopus, Embase (Ovid^®^ interface), and Google Scholar to identify population-based studies published from 1 January 1995 to 31 August 2021. In addition, PROSPERO was searched for any ongoing or recently completed systematic review of the topic. A combination of Boolean operators (AND, OR, and NOT), Medical Subject Headings (MeSH), truncation (*), and text words were used to search titles and abstracts using keywords such as “hypertension“, “high blood pressure”, “elevated blood pressure”, “prevalence”, “awareness”, “treatment”, “control”, “adults”, and “United Arab Emirates.” A detailed list of MeSH terms and keywords used for each database are presented in Supplemental Table S1.

### 2.2. Study Selection

Two researchers independently screened the titles and abstracts based on predefined inclusion criteria. All the collected articles were entered into EndNote reference Manager Software version 20 (Thomson Reuters, Stamford, CT, USA) to identify and remove duplicate records. Due to variations in reference studies from various sources, some references were manually screened. The collected articles were independently screened for their eligibility, and studies fulfilling the eligibility criteria were considered for full-text review.

#### 2.2.1. Inclusion Criteria

- Population-based prospective observational studies, conducted among apparently healthy adult subjects living in the UAE and that defined hypertension using the guidelines-recommended BP cut-off ≥130/80 mmHg or ≥140/90 mmHg, were considered.
- Studies provided estimates of the prevalence of hypertension and investigated the level of awareness, treatment, and control of hypertension among the general population.
- Multi-country studies were included if data on the prevalence, awareness, treatment, and control of hypertension in the UAE could be distinctly extracted.
- Only peer-reviewed full-length research articles were considered.

#### 2.2.2. Exclusion Criteria

- Studies conducted on diseased populations, children, and pregnant women were excluded.
- Studies that did not provide the estimates in numbers or percentages were excluded.
- Conference proceedings, abstracts, reviews, non-human studies, correspondences, and editorials were excluded.
- Studies with unrelated outcome measures and articles with missing or insufficient data were excluded.

### 2.3. Operational Definitions

- Prevalence of hypertension is defined as mean SBP ≥ 140 mmHg and/or diastolic BP (DBP) ≥ 90 mmHg and/or using antihypertensive medication if hypertension was known.
- Awareness of hypertension is defined as the proportion of subjects with hypertension who reported either having been diagnosed with hypertension by a clinician or reported taking antihypertensive medications.
- Hypertension treatment was defined as the proportion of adults with hypertension who reported taking any medication for hypertension.
- Hypertension control was defined as the proportion of adults taking antihypertensive medications but who had not reached the guidelines-recommended BP targets of <130/80 mmHg or <140/90 mmHg.

### 2.4. Data Extraction

Following the study protocol, two researchers independently screened titles and abstracts based on the eligibility criteria. Data, such as (1) authors’ names, data collection, and publication details, (2) study characteristics (study location, study design, type of settings, target population, sample size, mean age of the sample), (3) the type of device used to measure BP and the evaluation criteria, and (4) the outcome variables such as hypertension prevalence, awareness, treatment, and control, were collected.

### 2.5. Quality Assessment

The Newcastle-Ottawa Scale (NOS) was used to assess the methodological quality and risk of bias, and each included study was evaluated. The 7-item tool evaluated the quality of studies in three dimensions: (1) selection (4 items with one point allotted for each item: sample representativeness (1 point), sample selection procedure (1 point), exposure definition (1 point), and method of assessment (1 point)); (2) comparability (2 points); and (3) outcomes (assessment of outcomes (2 points) and statistical tests (1 point)). In accordance with the NOS scale, a maximum of nine points can be awarded to each study. An aggregated NOS score of six or more was considered high quality, whereas 0–5 indicated low quality.

### 2.6. Statistical Analysis

The estimates of the prevalence, awareness, treatment, and control of hypertension were expressed as proportions (%) with a corresponding 95% confidence interval (CI). The pooled prevalence estimates of each outcome variable were calculated using population size weights. The heterogeneity between the studies was assessed using the *I*^2^ statistics (% residual variation due to heterogeneity), and Tau^2^ (𝜏^2^) was used for each pooled estimate. The *I*^2^ values range between 0 and 100% and are considered as low heterogeneity for *I*^2^ < 25%, moderate for 25–50%, and high for >50%. When the heterogeneity was high, a random-effects DerSimonian–Laird model was used in the meta-analyses. In the case of substantial heterogeneity, the source of heterogeneity was investigated using stratified analyses and a meta-regression analysis based on various study-level characteristics. The interaction between the subgroups of each factor was evaluated using the Cochran *Q* test, the degree of freedom (df), and the *p*-value results for the Cochran *Q* test. Funnel plots were used to assess the publication bias assessment. Egger’s regression and Begg’s correlation tests were used to assess the statistical significance of publication bias. The statistical analyses were performed using STATA software version 16 MP (StataCorp, College Station, TX, USA). A *p*-value of <0.05 was considered statistically significant.

## 3. Results

### 3.1. Study Selection

**Figure 1:**
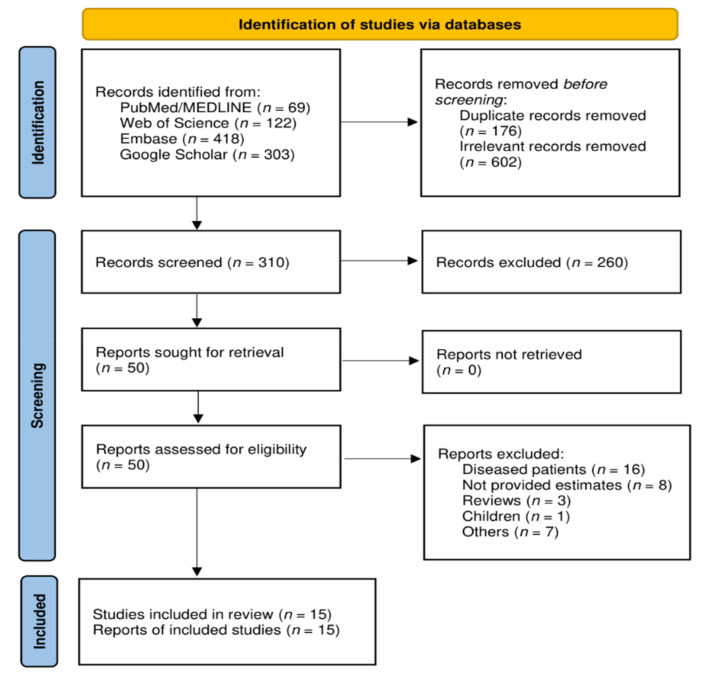
PRISMA Flow chart.

A total of 912 references were initially identified through electronic databases. After removing 176 duplicates and 602 irrelevant articles through the EndNote (Clarivate, Philadelphia, PA, USA) reference manager, a total of 310 records were screened using their titles and abstracts. Then, a full-text assessment of 50 potentially relevant articles resulted in 15 studies that fulfilled the eligibility criteria and that were included in the systematic review and meta-analysis [7,8,10,13,14,15,20,21,22,23,24,25,26,27,28] (Figure 1). Articles excluded for several reasons are shown in Supplemental Table S2.

### 3.2. Characteristics of the Included Studies

All the studies included in the study were published between 1995 [28] and 2020 [14]. Sample sizes range from 74 [24] to 50,138 [13], totaling 139,907 participants. All the studies were cross-sectional [7,8,10,13,14,15,20,21,22,23,24,25,26,27,28] and were conducted in community settings [7,8,10,13,22,23,25,27,28]. The majority of the studies were conducted in the general public [7,8,10,13,14,20,22,23,24,25,27,28], and some studies exclusively on South Asian immigrants [15] and men [21], including UAE citizens [26]. Moreover, most of the studies used manual [14,21,22,24,25,27,28] or automated [7,8,10,13,14,15,23,26] BP devices and defined hypertension as >130/80 mmHg [20], ≥135/85 mmHg [26], or ≥140/90 mmHg [7,8,10,13,14,15,23,24,25,27,28]. Overall, the mean age of the study population was 37.2 ± 8.7 years, and the studies reported a prevalence of hypertension ranging from 9.2% [21] to 52% [7]. More details are provided in Table 1.

### 3.3. Quality of Included Studies

The methodological quality assessment of the included studies was assessed using NOS for the cross-sectional studies. The average score of the NOS scale was 7.1 (range: 3–9). Overall, two studies were of low quality, with a NOS score of 0–5, and these studies were of a lower quality based on criteria 1 and 2 (sample selection and representativeness), criteria 5 (not report the definition of the exposure), and the appropriate statistical tests (criteria 7). Detailed results on the NOS quality assessment are presented in Supplemental Table S3.

### 3.4. Prevalence of Hypertension

**Figure 2:**
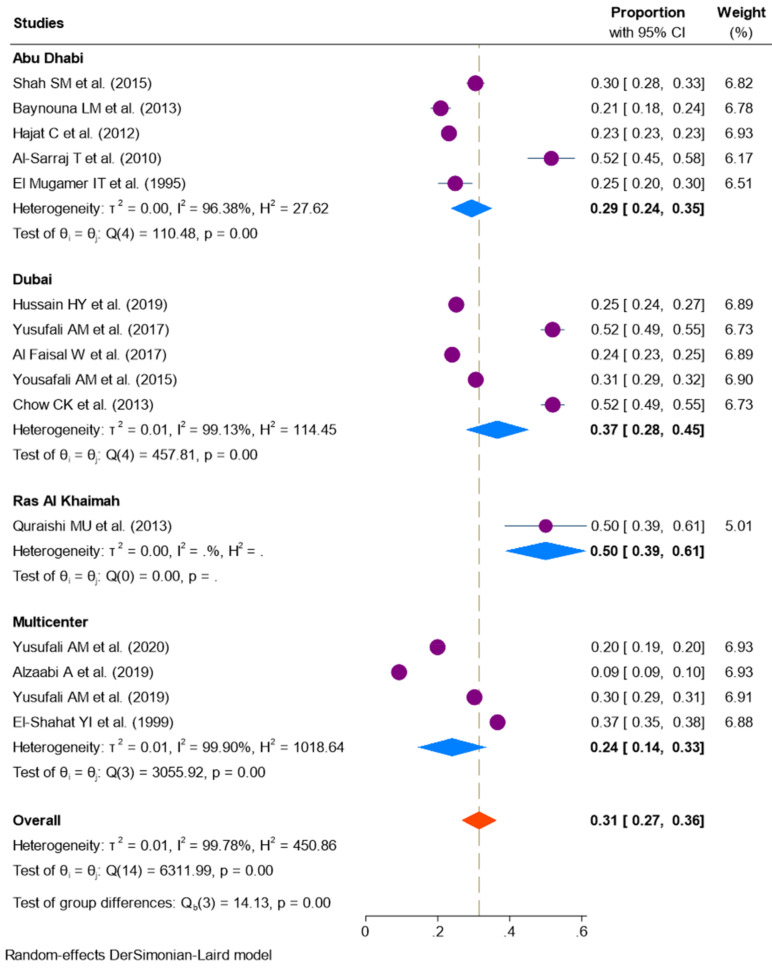
Prevalence of hypertension in the United Arab Emirates.

A total of fifteen studies [7,8,10,13,14,15,20,21,22,23,24,25,26,27,28], comprising 139,907 participants, reported a prevalence of hypertension in the UAE population. The distribution of hypertension across different emirates is shown in Figure 2. The pooled prevalence of hypertension in the UAE, after weighting the regional population size, was 31% (95% CI: 27–36; *I*^2^ = 99.7%, *p* < 0.001, τ^2^ = 0.01). Region-wise data showed significant differences in the prevalence of hypertension between each emirate in the UAE (*Q* = 14.1; df: 3; *p* < 0.001) ranging from 24% (95% CI: 14–33) in multicentered studies to 50% (95% CI: 39–61) in Ras Al Khaimah. Studies from Abu Dhabi and Dubai reported a pooled prevalence of 29% (95% CI: 24–35) and 37% (95% CI: 28–45), respectively.

### 3.5. Awareness, Treatment, and Control of Hypertension

**Figure 3:**
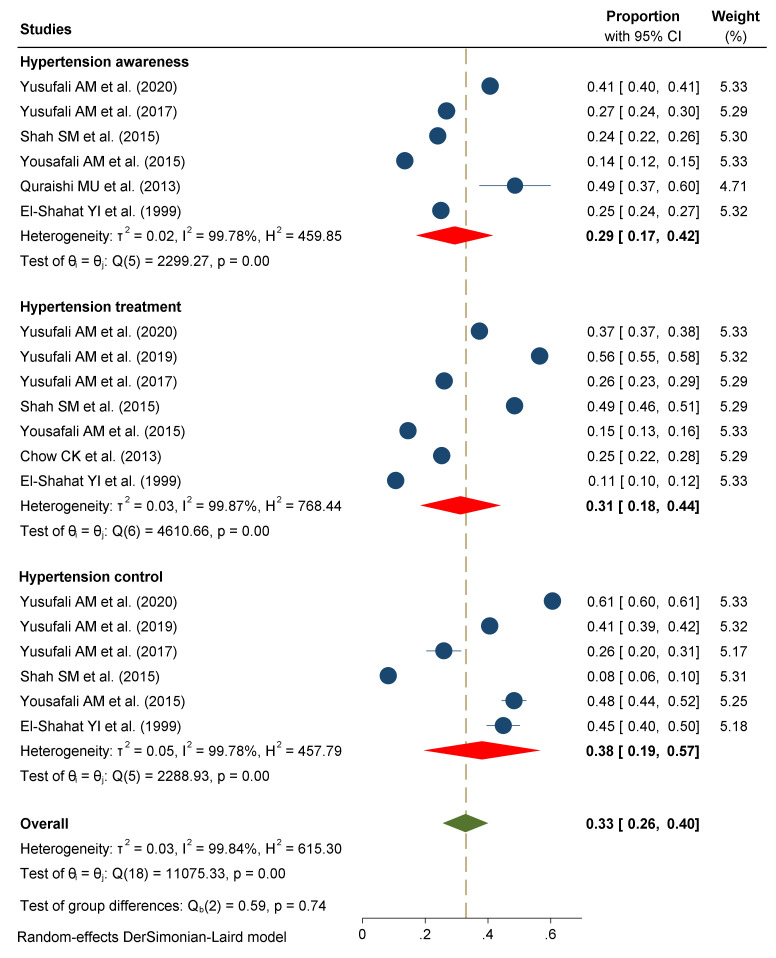
Hypertension awareness, treatment, and control in the United Arab Emirates.

The overall level of hypertension awareness, treatment, and control in the UAE was 33% (95% CI: 26–40; *I*^2^ = 99.8%). Six studies, comprising 40,906 participants, reported an awareness about hypertension in the UAE population [8,14,15,23,24,27]. The pooled estimates showed the overall level of awareness was 29% (95% CI: 17–42; *I*^2^ = 99.7; *p* < 0.001; τ^2^ = 0.02). Hypertension treatment in seven studies [7,8,14,15,23,24,27] was 11% to 56%, and the overall prevalence in 41,874 members of the UAE population with hypertension was 31% (95% CI: 18–44; *I*^2^ = 99.8; *p* < 0.001; τ^2^ = 0.03). Hypertension control in six studies [8,14,15,23,24,27] was between 8% and 61%, and the pooled prevalence in 40,960 hypertensive people under treatment was 38% (95% CI: 19–57; *I*^2^ = 99.7%; *p* < 0.01; τ^2^ = 0.05), as shown in Figure 3. However, there were no significant differences between the level of hypertension awareness, treatment, and control in the UAE (*Q* = 0.59; df = 2; *p* = 0.74).

### 3.6. Stratified Analysis

A stratified meta-analysis of the prevalence of hypertension in the UAE is summarized in Table 2. The prevalence of hypertension among the younger population (≤40 years) was 23% (95% CI: 16–30), among people of Arabian descent was 26% (95% CI: 22–29), among Emirati nationals was 27% (95% CI: 20–35), and among the South Asian population was 27% (95% CI: 24–31). We stratified the studies based on various baseline characteristics and interrogated the source of heterogeneity and the differences between the subgroups. Significant heterogeneity was observed among all the subgroups; for instance, studies comprising ≤1000 participants reported a higher prevalence than those comprising >1000 subjects. A significant heterogeneity was observed between the groups (*Q* = 4.54; df = 1; *p* = 0.03). Moreover, there were significant differences in the hypertension prevalence based on the type of device used to assess BP. Studies that assessed BP using an automated BP apparatus reported a higher prevalence of hypertension (38%, 95% CI: 32–45) than studies that used a manual BP device (25%, 95% CI: 19–32). Grouping the studies by various subgroups did not reduce heterogeneity, and no significant differences were observed between the groups (year of publication, percentage of females, type of health setting, and quality of studies).

### 3.7. Subgroup Analysis

Subgroup analysis by geographic region, study setting, type of BP apparatus, study population, and population characteristics significantly influenced hypertension prevalence, treatment, and control, as shown in Table 3. Interestingly, the younger population (≤40 years) had a higher awareness of hypertension (43%, 95% CI: 36–49) and BP control (61%, 95% CI: 60–61). However, lower hypertension control was observed in immigrant men (8%, 95% CI: 6–10).

### 3.8. Publication Bias

A visual examination of the funnel plots showed asymmetry and suggested that there is a source of publication bias, as shown in Supplemental Figure S1. In addition, the Egger test indicated a statistically significant publication bias for the hypertension prevalence estimates (Egger’s test *p* = 0.017).

## 4. Discussion

Hypertension is a major and often preventable risk factor for stroke, ischemic heart disease, heart failure, other vascular diseases, and renal disease worldwide [29]. Recent data comparing the hypertension prevalence, detection, treatment, and control covering all countries worldwide from 1990 to 2019 showed a higher age-standardized prevalence of hypertension 33% (95% CI: 31–36) and identified a considerable variation in hypertension control among those who were treated [30]. In particular, the study reported that the prevalence of hypertension in the UAE is higher in men (43.9%, 95% CI: 35.4–52.9) than in women (34.5%, 95% CI: 26.9–42.4), and the control was only 18.3% (95% CI: 9.9–29.2) in men, compared to 24.6% (95% CI: 13.2–38.6) in women. However, there is no nationally representative study of the burden, treatment, and control of hypertension in the UAE. Several small studies have been conducted in different parts of the UAE and have reported on hypertension prevalence, awareness, treatment, and control. Therefore, this systematic synthesis of the available data provided comprehensive evidence of hypertension in the UAE.

To the authors’ knowledge, this is the first comprehensive assessment on the prevalence, awareness, treatment, and control of hypertension in the UAE. Data from fifteen studies, comprising 139,907 participants in the UAE over the last 25 years, showed a higher prevalence of hypertension (31%), awareness (29%), treatment (31%), and control (38%). Region-specific estimates showed that hypertension is widely prevalent in Dubai (37%) and Abu Dhabi (29%). A stratified analysis showed variations in hypertension prevalence across subsets, and a higher prevalence was observed in the general public (33%) and community settings (33%) and was identified through automated BP devices (38%). Furthermore, there were considerable variations in hypertension awareness, treatment, and control across subgroups.

We carefully assessed the methodological quality of studies using accepted quality scores. There were very few low-quality studies. All the included studies used a cross-sectional design and were conducted on the UAE’s representative population. Region-wise variations in the prevalence of hypertension noted in this study could be due to the inclusion of a limited number of studies with a smaller sample size, the BP criteria used for diagnosis of hypertension, gender differences, study settings, and variations in the BP measurement devices. Moreover, most of the studies were conducted in cities such as Abu Dhabi and Dubai, where the hypertension prevalence and level of awareness were very high compared to other emirates. However, only one study from Ras Al Khaimah [24] with a smaller sample size (*n* = 74) was identified, and it reported that half of the participants were hypertensives. To overcome these discrepancies, nationwide population-representative studies are needed to understand the burden of hypertension in the UAE population.

To benchmark hypertension prevalence, awareness, treatment, and control in the UAE, pooled estimates from this study were compared to country-specific estimates of the NCD-RisC study published in Lancet 2021 [30]. Country-specific data from the Arabian Gulf countries showed that the 31% prevalence of hypertension in the UAE is lower than that in Saudi Arabia (33.3%), Bahrain (37.7%), Kuwait (39.4%), Qatar (39.7%), and Oman (43.6%) [30]. However, the UAE data presented in the Non-Communicable Disease NCD Risk Factor Collaboration (NCD-RisC) study reported that the prevalence of hypertension in the UAE was 39.2% [31], which is higher than our pooled estimates (31%). Differences in the health survey data, diagnoses and treatments of hypertension using questionnaires, BP measurement errors, validation of the BP devices, and several other residual factors may contribute to the differences in estimating the prevalence of hypertension.

Comparing the level of hypertension awareness, treatment, and control to data from other high-income countries [5] showed that the level of awareness in the UAE (29%) was much lower than in Australia (74%), Canada (83%), Germany (87%), Japan (70%), New Zealand (74%), South Korea (79%), the United Kingdom (72%), and the United States of America (USA) (84%). Similarly, the hypertension control in the UAE (38%) that was observed in this study was lower than in Canada (66%), Germany (53%), South Korea (50%), and the USA (50%), but higher than in Australia (33%), New Zealand (31%) and Japan (25%) [5]. Variations in the combination of the enabling factors of high prevalence, low awareness, and poor hypertension control calls for an urgent response in line with Sustainable Development Goals (SDG) target 3.4 on Non-communicable Diseases (NCDs) to lower hypertension prevalence or control BP through both improved prevention and improving early-stage treatment cascades [2,31]. The changing prevalence of hypertension in the UAE is mainly due to changing lifestyles with lower physical activity and a shift from a traditional diet that is high in fiber to energy-dense processed food high in fat, sugar, and salt [32,33,34]. Furthermore, studies consistently reported a significantly increased burden of overweight and obesity [35], metabolic syndrome [36], and cardiovascular risk factors [37] among the younger population in recent decades. Therefore, public health authorities should initiate multifaceted interventions to control the pervasive burden of hypertension in the UAE.

### Strengths and Limitations

There are several strengths and some limitations in this study. This systematic review and meta-analyses consolidated the quantitative evidence on the prevalence, awareness, treatment, and control of hypertension in the UAE from 1995 to 2021. We employed a comprehensive search strategy across several data sources, involved many study participants in providing pooled estimates, and thoroughly assessed the risk of bias in each of the 15 observational studies. Furthermore, we conducted stratified meta-analyses to investigate the potential source of heterogeneity between the studies and subgroups.

Similar to all systematic reviews and meta-analyses, our study has some limitations. First, baseline characteristics, such as geographic area, differences in the culture, BP assessment methods, health settings, population characteristics, and practices, vary widely across the UAE, which might influence our results. Second, the outcomes reported in this study were obtained from cross-sectional studies, and thus are not conclusive to generalize to the UAE population. Third, high heterogeneity was observed across all of the outcomes; this might be due to several underlying reasons that warrant further investigation; we performed a stratified analysis and a subgroup analysis to investigate the source of heterogeneity. Fourth, low power and precision may have contributed to higher heterogeneity (Cochran *Q*) and *I^2^*. Fifth, the Egger test suggested a publication bias in the pooled prevalence of hypertension in the UAE. Thus, caution is needed when interpreting the findings.

## 5. Conclusions

Our findings indicated that a significant prevalence of hypertension, poor awareness, and suboptimal BP control was observed in the UAE—nearly one in three adults have hypertension while one in five control it. Significant regional differences exist in hypertension prevalence and care in the UAE. These findings highlight the urgent need for multifaceted interventions that include the early screening for and detection of BP—particularly in high-risk populations—raising awareness, and improving hypertension diagnoses and treatments.

## Figures and Tables

**Table 1:** Characteristics of the included studies.

Author, Year	Study Characteristics						BP Device	Evaluation Criteria	Prevalence (%)			
	Location	Study Design	Setting	Population	Screened Population	Mean Age			Hypertension	Awareness	Treatment	Control
Yusufali A. et al. (2020) [14]	Seven emirates	CS	Health centers	General public	31,316	36.8 (11.4)	Automated /Manual	≥140/90 mmHg	19.9	40.7	37.3	60.6
Hussain H.Y. et al. (2019) [20]	Dubai	CS	Household	General public	3289		Manual	>130/80 mmHg	25.1	-	-	-
Alzaabi A. et al. (2019) [21]	Abu Dhabi, Al Ain, Sharjah	CS	Medical examination centers	Men	33,327	21.6	Manual	≥140/90 mmHg	9.2	-	-	-
Yusufali A. et al. (2019) [10]	Seven emirates	CS	Community	General public	6193	38.2 (13.1)	Automated	≥140/90 mmHg	30.2	-	56.5	40.6
Yusufali A.M. et al. (2017) [8]	Dubai	CS	Community	General public	917	49.5 (10.3)	Automated	≥140/90 mmHg	51.9	26.8	26.1	25.9
Al Faisal W. et al. (2017) [22]	Dubai	CS	Community	General public	3716		Manual	≥140/90 mmHg	24.0	-	-	-
Shah S.M. et al. (2015) [15]	Al Ain	CS	Community	South Asian Immigrants	1375	34	Automated	≥140/90 mmHg	30.5	24.0	48.5	8.2
Yusufali A.M. et al. (2015) [23]	Dubai	CS	Community, hospital	General public	4128	38.4 (11.4)	Automated	≥140/90 mmHg	30.5	13.5	14.5	48.3
Quraishi M.U. et al. (2013) [24]	Ras Al Khaimah	CS	Kerala market	General public	74		Manual	≥140/90 mmHg	50.0	48.6	-	-
Baynouna L.M. et al. (2013) [25]	Al Ain	CS	Community	General public	817		Manual	≥140/90 mmHg	20.8	-	-	-
Chow C.K. et al. (2013) [7]	Dubai	CS	Community	General public	918	49.1 (10.2)	Automated	≥140/90 mmHg	52.0	-	25.3	-
Hajat C. et al. (2012) [13]	Abu Dhabi	CS	Community	General public	50,138	36.8 (14.3)	Automated	≥140/90 mmHg	23.1	-	-	-
Al-Sarraj T. et al. (2010) [26]	Al Ain	CS	Hospital	UAE citizens	227	31.2 (8.9)	Automated	≥135/85 mmHg	51.5	-	-	-
El-Shahat Y.I. et al. (1999) [27]	Abu Dhabi, Al Ain, Sharjah	CS	Community	General public	3150		Manual	≥140/90 mmHg	36.6	25.0	10.6	44.9
el Mugamer I.T. et al. (1995) [28]	Abu Dhabi	CS	Community	General public	322		Manual	≥140/90 mmHg	25.2	40.7	37.3	60.6

CS: cross-sectional design.

**Table 2:** Stratified meta-analysis of the prevalence of hypertension in the United Arab Emirates.

Characteristics	Number of Studies	Pooled Prevalence in Percentage (95% CI)	*p* for Interaction ^†^	*I*^2^ (%)	Z	Heterogeneity between Groups		
						*Q*	*df*	*p*
Year			0.096			2.80	1	0.09
1995–2015	9	35 (2941)		98.9	11.5			
2016–2020	6	27 (1934)		99.8	6.84			
Screened population			0.001			4.54	1	0.03
≤1000	6	42 (28–55)		98.5	5.92			
>1000	9	25 (20–31)		99.8	8.72			
Type of population			0.008					
General public	13	33 (29–36)		99.1	18.8			
Men only	2	10 (9–10)		-	60.9			
Percent of female			0.083			3.25	1	0.07
<50%	6	39 (29–50)		99.4	7.22			
≥50%	3	27 (19–35)		99.5	6.51			
Type of setting			0.387			2.15	2	0.34
Community	11	33 (28–37)		98.9	14.1			
Hospital	3	26 (16–35)		-	5.50			
Both	1	31 (29–32)		-	42.60			
Type of device			0.006			7.50	1	0.01
Manual	8	25 (19–32)		99.7	7.85			
Automated	7	38 (32–45)		99.3	11.38			
Sub-population								
Age ≤ 40 years	5	23 (16–30)	-	99.7	6.33	7.38	1	0.01
Arab descents	5	26 (22–29)	-	99.1	15.37	2.63	1	0.10
Emirati	8	27 (20–35)	-	98.8	7.36	2.95	1	0.09
South Asian	7	27 (24–31)		98.8	17.61	0.96	1	0.33
Quality of studies			0.738			0.06	1	0.81
High (> 7 score)	6	33 (25–38)		99.5	7.13			
Low (≤ 7 score)	9	31 (23–38)		99.9	12.43			

^†^*p*-value from meta-regression analyses.

**Table 3:** Subgroup analysis for the potential variables between the studies of Hypertension awareness, treatment, and control in the United Arab Emirates.

Subgroup		No. of Studies	Proportion (95% CI)	Test for Heterogeneity		Between Subgroup Differences		
				*Tau^2^*	*I^2^*	*Q*	*df*	*p*
**Hypertension Awareness**								
Geographic region	Abu Dhabi	1	24 (22–26)	-	-	18.8	3	0.001
	Dubai	2	20 (7–33)	0.01	98.6			
	Ras Al Khaimah	1	49 (37–60)	-	-			
	Multicenter	2	33 (18–48)	0.01	99.7			
Study setting	Community	4	27 (23–31)	0.00	83.9	2069.9	2	0.001
	Healthcare	1	41 (40–41)	-	-			
	Both	1	14 (12–15)	-	-			
Type of BP device	Manual	2	25 (24–27)	0.03	93.8	0.17	1	0.68
	Automated	4	29 (12–46)	0.03	99.8			
Study population	General public	5	31 (16–45)	0.03	99.8	0.80	1	0.37
	Immigrant men	1	24 (22–26)	-	-			
Population characteristics	Age ≤ 40 years	2	43 (36–49)	0.00	46.4	1.87	1	0.17
	Nationals	2	19 (8–31)	0.01	99.3	-	-	-
	Expatriates	4	31 (14–49)	0.03	99.8	2142.6	3	0.001
**Hypertension Treatment**								
Geographic region	Abu Dhabi	1	49 (46–51)	-	-	45.0	2	0.001
	Dubai	3	22 (14–29)	0.00	97.0			
	Multicenter	3	35 (9–61)	0.05	99.9			
Study setting	Community	5	33 (17–50)	0.04	99.7	1379.8	2	0.001
	Healthcare	1	37 (37–38)	-	-			
	Both	1	15 (13–16)	-	-			
Type of device	Manual	1	11 (10–12)	0.00	-	15.78	1	0.001
	Automated	6	36 (24–49)	0.03	99.8			
Study population	General public	6	28 (15–42)	0.03	99.8	8.36	1	0.001
	Immigrant men	1	49 (46–51)	-	-			
Population characteristics	Age ≤ 40 years	1	37 (37–38)	-	-			
	Nationals	2	13 (9–16)	0.00	96.1	25.6	1	0.001
	Expatriates	4	39 (21–57)	0.03	99.9	2697.6	3	0.001
**Hypertension control**								
Geographic region	Abu Dhabi	1	8 (6–10)	-	-	47.9	2	0.001
	Dubai	2	37 (15–59)	0.02	97.5			
	Multicenter	3	49 (37–61)	0.01	99.2			
Study setting	Community	4	30 (14–46)	0.03	99.2	47.4	2	0.001
	Healthcare	1	61 (60–61)	-	-			
	Both	1	48 (44–52)	-	-			
Type of device	Manual	1	45 (40–50)	-	-	0.55	1	0.46
	Automated	5	37 (16–58)	0.06	99.8			
Study population	General public	5	44 (33–55)	0.02	99.1	39.6	1	0.001
	Immigrant men	1	8 (6–10)	-	-			
Population characteristics	Age ≤ 40 years	1	61 (60–61)	-	-			
	Nationals	2	47 (44–50)	0.00	1.27	1.01	1	0.31
	Expatriates	4	39 (17–61)	0.05	99.8	2214.3	3	0.001

CI: confidence interval.

## Data Availability

Data sharing not applicable.

## Supplementary Materials

The following are available online at https://www.mdpi.com/article/10.3390/ijerph182312693/s1, Table S1: Search strategy, Table S2: Articles excluded with reason, Table S3: Quality assessment of included studies using Newcastle-Ottawa scale adapted for cross-sectional studies, Figure S1: Funnel plots.
