# Supplementary material for: Prevalence, Awareness, Treatment, and Control of Hypertension in the United Arab Emirates: A Systematic Review and Meta-Analysis
Source: Int J Environ Res Public Health. 2021 Dec 2;18(23):12693. doi: 10.3390/ijerph182312693 (PMC8657061; doi:10.3390/ijerph182312693)
Supplement: Supplementary file 1 [file ijerph-18-12693-s001.zip › ijerph-1431723-supplementary.pdf]

Supplement files

Table S1. Search strategy

PubMed/MEDLINE	SCOPUS	Embase	Google scholar
“(((((((((prevalence) OR disease burden) OR estimate) OR trend)) AND ((((((((((((hypertension) OR blood pressure) OR raised blood pressure) OR elevated blood pressure) OR systolic blood pressure) OR diastolic blood pressure) OR SBP) OR DBP) OR isolated systolic blood pressure) OR high BP) OR BP) OR raised BP) OR elevated BP))) OR ((((((((risk factors) OR awareness) OR control) AND blood pressure)) OR ((((risk factors) OR awareness) OR control) AND hypertension))) AND ((((((((((((hypertension) OR blood pressure) OR raised blood pressure) OR elevated blood pressure) OR systolic blood pressure) OR diastolic blood pressure) OR SBP) OR DBP) OR isolated systolic blood pressure) OR high BP) OR BP) OR raised BP) OR elevated BP)))) AND (United Arab Emirates))”.	(ALL (hypertension) OR TITLE-ABS-KEY (hypertension) AND ALL (blood AND pressure) OR TITLE-ABS-KEY (blood AND pressure) AND ALL (elevated AND blood AND pressure) OR TITLE-ABS-KEY (elevated AND blood AND pressure) AND ALL (systolic AND blood AND pressure) OR TITLE-ABS-KEY (systolic AND blood AND pressure) AND ALL (diastolic AND blood AND pressure) OR TITLE-ABS-KEY (diastolic AND bp) AND ALL (awareness) OR TITLE-ABS-KEY (awareness) AND ALL (control) OR TITLE-ABS-KEY (control) AND TITLE-ABS-KEY (knowledge) AND ALL (knowledge) AND ALL (treatment) OR TITLE-ABS-KEY (treatment) AND ALL (united AND arab AND emirates) AND TITLE-ABS-KEY (uae) AND ALL (emirates))	(hypertension OR hypertension:ti OR hypertension:ab,ti OR hypertension:ti,ab,kw OR hypertension:kw OR hypertension:ab) AND 'united arab emirates'. ('elevated blood pressure' OR 'blood pressure':ti OR 'systolic blood pressure':ab,ti OR 'blood pressure measurement':ti,ab,kw OR 'blood pressure':kw OR 'blood pressure measurement':ab) AND 'united arab emirates' AND 'cross-sectional study' AND [1995-2021]/py. (hypertension:ti OR awareness OR 'awareness questionnaire':ti OR knowledge:ab,ti OR awareness:ti,ab,kw OR awareness:kw OR 'awareness questionnaire':ab) AND 'united arab emirates' AND 'cross-sectional study' AND [1995-2021]/py. (hypertension:ti OR control OR 'awareness questionnaire':ti OR treatment:ab,ti OR awareness:ti,ab,kw OR control:kw OR treatment:ab) AND 'united arab emirates' AND	“prevalence, hypertension, awareness, screening, blood pressure, elevated systolic blood pressure, control, treatment, united arab emirates Hypertension OR blood OR pressure "hypertension OR blood pressure OR elevated blood pressure OR screening" prevalence Hypertension OR blood pressure OR elevated BP OR blood OR pressure OR elevated diastolic OR elevated systolic OR elevated OR blood OR pressure OR elevated OR systolic OR blood OR pressure OR elevated BP OR elevated hypertension OR BP "hypertension OR blood pressure OR elevated blood pressure OR screening NOT " -gestation -OR -children”

		'cross-sectional study' AND ('control'/exp OR control) AND [1995-2021]/py	

Table S2: Articles excluded with reason

1. Alkaabi MS, Rabbani SA, Rao PG, Ali SR. Evaluation of antihypertensive prescriptions for rationality and adherence to treatment guidelines: An experience from United Arab Emirates. Clinical Epidemiology and Global Health. 2020 Sep 1;8(3):764-9. (Hypertensive patients)

2. Mussa BM, Abduallah Y, Abusnana SJ. Prevalence of hypertension and obesity among emirati patients with type 2 diabetes. J Diabetes Metab. 2016 Jan 1;7(1):1-5. (T2DM patients)

3. Basha SA, Mathew E, Sreedharan J, Muttappallymyalil J, Sharbatti SA, Shaikh RB. Pattern of Blood Pressure Distribution among University Students in Ajman, United Arab Emirates. Nepal Journal of Epidemiology. 2011 Dec 6;1(3):86-9. (Not provided the prevalence estimates)

4. Mathew E, Ahmed M, Hamid S, Abdulla F, Batool K. Hypertension and Dyslipidemia in Type 2 Diabetes Mellitus in United Arab Emirates. Australasian Medical Journal. 2010 Nov 1;3(11). (T2DM patients)

5. Alomar MJ, Strauch CC. A prospective evaluation of antihypertensive medications safety and efficacy in United Arab Emirates private hospitals. Am. J. Pharmacol. Toxicol. 2010;5(2):89-94. (Hypertensive inpatients).

6. Baynouna LM, Neglekerke NJ, Ali HE, ZeinAlDeen SM, Al Ameri TA. Audit of healthy lifestyle behaviors among patients with diabetes and hypertension attending ambulatory health care services in the United Arab Emirates. Global health promotion. 2014 Dec;21(4):44-51. (HTN and DM patients).

7. Radaideh G, Tzemos N, Ali TM, Eldershaby Y, Joury J, Abreu P. Cardiovascular Risk Factor Burden in the United Arab Emirates (UAE): The Africa Middle East (AfME) Cardiovascular Epidemiological (ACE) Study Sub-analysis. International Cardiovascular Forum Journal 2017 Jun 3 (Vol. 11). (not stratified the UAE population).

8. Abdulle AM, Nagelkerke NJ, Abouchacra S, Pathan JY, Adem A, Obineche EN. Under-treatment and under diagnosis of hypertension: a serious problem in the United Arab Emirates. BMC cardiovascular disorders. 2006 Dec;6(1):1-7. (Hypertension patients)

9. Sabri S, Bener A, Eapen V, Abu Zeid MS, Al Mazrouei AM, Singh J. Some risk factors for hypertension in the United Arab Emirates. EMHJ-Eastern Mediterranean Health Journal, 10 (4-5), 610-619, 2004. 2004. (Hypertension patients).

10. Al-Sharbatti S, Shaikh R, Mathew E, Sreedharan J, Muttappallymyalil J, Basha S. The Use of Obesity Indicators for the Prediction of Hypertension Risk among Youth in the United Arab Emirates. Iran J Public Health. 2011;40(3):33-40. (Not provided prevalence estimates).

11. Sabri S, Bener A, Eapen V, Azhar AA, Abdishakure A, Singh J. Correlation between hypertension and income distribution among United Arab Emirates population. Medical Journal of Malaysia. 2005 Oct 1;60(4):416. (Not provided prevalence estimates)

12. Malik M, Bakir A, Abi Saab B, Roglic G, King H. Glucose intolerance and associated factors in the multi-ethnic population of the United Arab Emirates: results of a national survey. Diabetes research and clinical practice. 2005 Aug 1;69(2):188-95. (Not provided prevalence estimates).

13. Baynouna LM, Nagelkerke NJ, Al Ameri TA, Al Deen SM, Ali HI. Determinants of diabetes and hypertension control in ambulatory healthcare in Al ain, United Arab Emirates. Oman medical journal. 2014 May;29(3):234. (Hypertension patients).

14. Shaikh RB, Mathew E, Sreedharan J, Muttappallymyalil J, Al Sharbatti S, Basha SA. Knowledge regarding risk factors of hypertension among entry year students of a medical university. Journal of family and community medicine. 2011 Sep;18(3):124. (Not related to this study objective).

15. Shantakumari N, Eldeeb RA, Ibrahim SA, Sreedharan J, Otoum S. Effect of PUFA on patients with hypertension: a hospital based study. indian heart journal. 2014 Jul 1;66(4):408-14. (hypertension patients)

16. Bader RJ, Koprulu F, Hassan NA, Ali AA, Elnour AA. Predictors of adherence to antihypertensive medication in northern United Arab Emirates. Eastern Mediterranean Health Journal. 2015 May 1;21(5). (hypertension patients).

17. Al-Kaabi J, Al-Maskari F, Saadi H, Afandi B, Parkar H, Nagelkerke N. Assessment of dietary practice among diabetic patients in the United Arab Emirates. The review of diabetic studies: RDS. 2008;5(2):110. (DM patients).

18. Alhyas L, McKay A, Balasanthiran A, Majeed A. Prevalences of overweight, obesity, hyperglycaemia, hypertension and dyslipidaemia in the Gulf: systematic review. JRSM short reports. 2011 Jul;2(7):1-6. (review article)

19. Tailakh A, Evangelista LS, Mentes JC, Pike NA, Phillips LR, Morisky DE. Hypertension prevalence, awareness, and control in A rab countries: A systematic review. Nursing & health sciences. 2014 Mar;16(1):126-30. (Review article)

20. Akl C, Akik C, Ghattas H, Obermeyer CM. Gender disparities in midlife hypertension: a review of the evidence on the Arab region. Women's midlife health. 2017 Dec;3(1):1-0. (Review article).

21. Frossard PM, Lestringant GG, Malloy MJ, Kane JP. Human renin gene BglI dimorphism associated with hypertension in two independent populations. Clinical genetics. 1999 Dec;56(6):428-33. (Genetic study)

22. Ahmad U, Saleheen D, Bokhari A, Frossard PM. Strong association of a renin intronic dimorphism with essential hypertension. Hypertension research. 2005 Apr;28(4):339-44. (Genetic study)

23. Frossard PM, Lestringant GG, Elshahat YI, John A, Obineche EN. An MboI two-allele polymorphism may implicate the human renin gene in primary hypertension. Hypertension Research. 1998;21(3):221-5. (Genetic study)

24. Abdulle A, Al-Junaibi A, Nagelkerke N. High blood pressure and its association with body weight among children and adolescents in the United Arab Emirates. PloS one. 2014 Jan 20;9(1):e85129. (Children)

25. Jelinek HF, Osman WM, Khandoker AH, Khalaf K, Lee S, Almahmeed W, Alsafar HS. Clinical profiles, comorbidities and complications of type 2 diabetes mellitus in patients from United Arab Emirates. BMJ Open Diabetes Research and Care. 2017 Aug 1;5(1). (T2DM patients)

26. Frossard PM, Obineche EN, Lestringant GG, Elshahat YI. Association study between the ANF gene and hypertension in a Gulf Arab population. American journal of hypertension. 1997 Nov 1;10(11):1308-10. (Genetic study)

27. Frossard PM, Hill SH, Elshahat YI, Obineche EN, Bokhari AM, Lestringant GG, John A, Abdulle AM. Associations of angiotensinogen gene mutations with hypertension and myocardial infarction in a gulf population. Clinical genetics. 1998 Oct;54(4):285-93. (Genetic study).

28. Saadi H, Carruthers SG, Nagelkerke N, Al-Maskari F, Afandi B, Reed R, Lukic M, Nicholls MG, Kazam E, Algawi K, Al-Kaabi J. Prevalence of diabetes mellitus and its complications in a population-based sample in Al Ain, United Arab Emirates. Diabetes research and clinical practice. 2007 Dec 1;78(3):369-77. (DM patients).

30. Akl C, Akik C, Ghattas H, Obermeyer CM. The cascade of care in managing hypertension in the Arab world: a systematic assessment of the evidence on awareness, treatment and control. BMC Public Health. 2020 Dec;20:1-3. (Review article).

31. Al-Kaabi J, Al-Maskari F, Afandi B, Parkar H, Nagelkerke N. Physical activity and reported barriers to activity among type 2 diabetic patients in the United Arab Emirates. The review of diabetic studies: RDS. 2009;6(4):271. (DM patients)

32. Sulaiman N, Elbadawi S, Hussein A, Abusnana S, Madani A, Mairghani M, Alawadi F, Sulaiman A, Zimmet P, Huse O, Shaw J. Prevalence of overweight and obesity in United Arab Emirates Expatriates: the UAE national diabetes and lifestyle study. Diabetology & metabolic syndrome. 2017 Dec;9(1):1-9. (Not provided estimates of hypertension)

33. Ibrahim OH, Jirjees F, Mahdi H. Barriers affecting compliance of patients with chronic diseases: a preliminary study in United Arab Emirates (UAE) population. Asian J Pharm Clin Res. 2011;4(2):42-5. (Not related to our study objective)

34. Al-Maskari F, El-Sadig M, Nagelkerke N. Assessment of the direct medical costs of diabetes mellitus and its complications in the United Arab Emirates. BMC Public Health. 2010 Dec;10(1):1-0. (Diabetes patients)

35. Tailakh A, Mentes JC, Morisky DE, Pike NA, Phillips LR, Evangelista LS. Prevalence, awareness, treatment, and control of hypertension among Arab Americans. The Journal of cardiovascular nursing. 2013 Jul;28(4):330. (Arab People living in USA)

Table S3: Quality assessment of included studies using Newcastle-Ottawa scale adapted for cross-sectional studies

Cross-sectional study	Selection				Comparability[5]	Outcome		Total
	Representativeness[1]	Selection [2]	Definition [3]	Assessment[4]		Assessment[6]	Statistical test[7]	
Yusufali A et al. (2020)	1	1	1	0	1	2	1	7
Hussain HY et al. (2019)	1	1	1	1	0	2	1	6
Alzaabi A et al. (2019)	1	1	1	1	2	1	1	8
Yusufali A et al. (2019)	1	1	1	1	1	2	1	8
Yusufali AM et al. (2017)	1	1	1	1	2	2	1	9
Al Faisal W et al. (2017)	1	1	1	1	1	1	1	7
Shah SM et al. (2015)	1	1	1	1	2	2	1	9
Yusufali AM et al. (2015)	1	1	1	1	1	1	1	7
Quraishi MU et al. (2013)	0	0	1	1	0	1	0	3
Baynouna LM et al. (2013)	1	1	1	1	1	1	1	7
Chow CK et al. (2013)	1	1	1	1	2	2	1	9
Hajat C et al. (2012)	1	1	1	1	2	2	1	9
Al-Sarraj T et al. (2010)	1	1	1	1	0	1	1	6
El-Shahat YI et al. (1999)	1	1	1	1	1	1	1	7
El Mugamer IT et al. (1995)	1	1	1	1	0	1	0	5

1: sample representativeness; 2: sample selection procedure; 3: exposure definition; 4: risk factor exposure assessment; 5: Study controls for the most important factor or any additional factor 6: Assessment of the outcome; 7: Statistical test is appropriate or not.

Figure S1: Funnel plots

Prevalence of Hypertension in the UAE	Hypertension awareness in the UAE
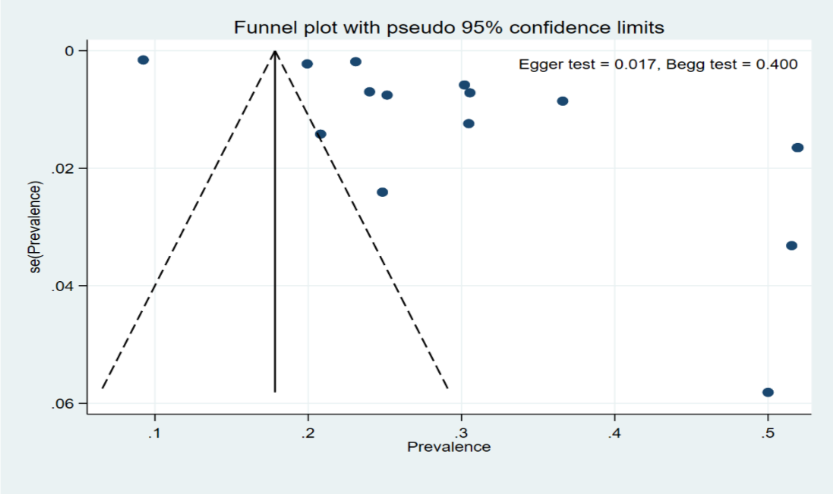	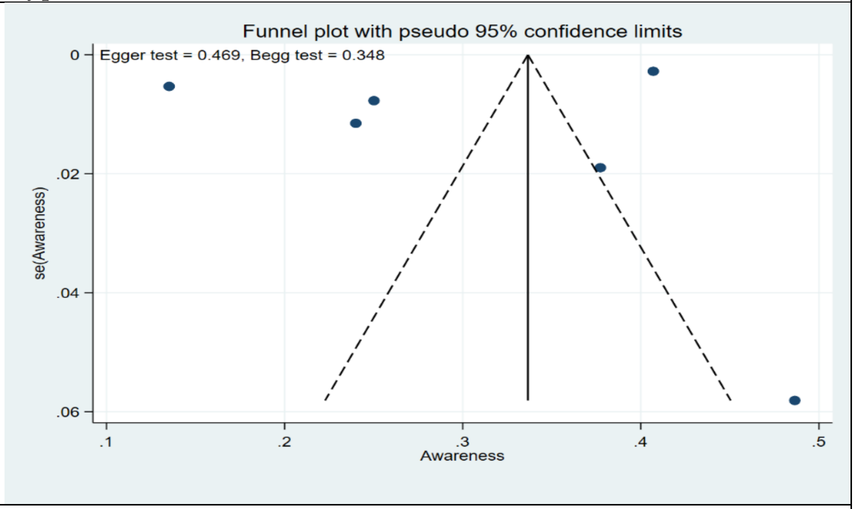
**Hypertension treatment in the UAE**	**Hypertension control in the UAE**
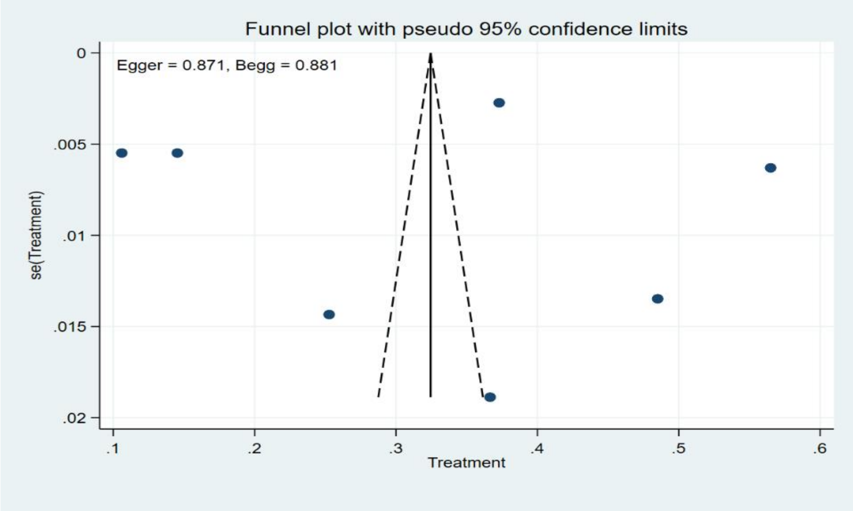	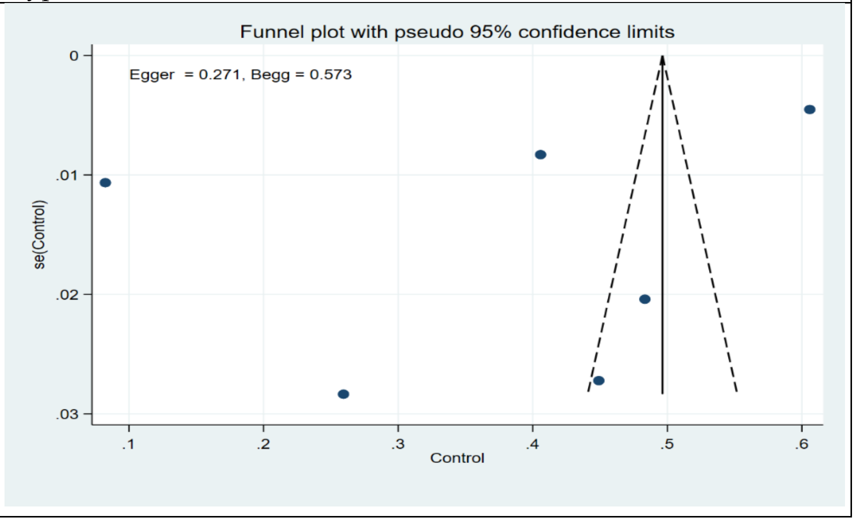
